# Evaluating magnetic resonance imaging characteristics and risk factors for hemifacial spasm

**DOI:** 10.1002/brb3.3438

**Published:** 2024-02-26

**Authors:** Bo Li, Chun Luo, Yabin Jin, Ying Yi, Dongliang Cheng, Linwen Huang, Guofu Wang, Xuguang Zhong, Hai Zhao, Mingyong Gao

**Affiliations:** ^1^ Department of Radiology The First People's Hospital of Foshan Foshan Guangdong Province China; ^2^ Sun Yat‐Sen University Guangzhou Guangdong Province China; ^3^ Institute of Translational Medicine The First People's Hospital of Foshan Foshan Guangdong Province China; ^4^ Department of Functional Neurosurgery The First People's Hospital of Foshan Foshan Guangdong Province China

**Keywords:** facial nerve, hemifacial spasm, magnetic resonance imaging, neurovascular compression

## Abstract

**Purpose:**

The specific neurovascular compression (NVC) event responsible for the symptomatic manifestation of hemifacial spasm (HFS) remains difficult to assess accurately using magnetic resonance imaging (MRI). We aim to evaluate the MRI characteristics of HFS.

**Method:**

We retrospectively included patients with HFS and divided them into a test group (*n* = 186) and a validation group (*n* = 28). The presence, severity, and offending vessel type of NVC in each portion, and the orientation of the offending vessel around the facial nerve, were recorded. Conditional logistic regression analyses were performed to evaluate correlations using test group. The validation group was used to verify whether our findings improved diagnostic performance.

**Results:**

Deformity in the proximal cisternal segment was significantly correlated with HFS occurrence (odds ratio [OR]: 256.58, *p* = .002), whereas contact was not (*p *= .233). Both contact and deformity in the root detachment point (OR: 19.98 and 37.22, *p* < .001 and *p* = .013, respectively) or attached segment (OR: 4.99 and 252.52, *p* = .001 and *p* < .001, respectively) were significantly correlated with HFS occurrence. Our findings improved specificity, positive predictive value, and accuracy of diagnosis than conventional diagnostic methods. The vertebral artery predominantly compress the facial nerve in the inferior‐anterior position, the anterior inferior cerebellar artery predominantly in the inferior position, the posterior inferior cerebellar artery predominantly in the inferior position, vein predominantly in the posterior–superior position.

**Conclusions:**

This study further demonstrates that within the susceptible portion of facial nerve, different portions of the nerve respond differently to NVC. Each offending vessel has its own preferred conflict orientation. Our study offers reference for neurosurgeons in diagnosis and treatment.

## Graphical abstract

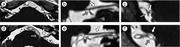

## 1 INTRODUCTION

Hemifacial spasm (HFS) is a movement disorder that affects the muscles innervated by the facial nerve, resulting in involuntary contractions of the facial muscles (Blitzer & Phelps, 2020). Although HFS is not lethal, it usually causes considerable cosmetic and functional disability, which has a profound impact on the physical and mental health in patients with HFS (Setthawatcharawanich et al., 2011). Neurovascular compression (NVC) of the facial nerve is the common cause of HFS. Microvascular decompression surgery, as the exclusive causal treatment, alleviates facial nerve compression by the offending vessel, thus providing a radical solution to the problem (Jannetta, 1975; Miller & Miller, 2012; Nugroho et al., 2021; Rosenstengel et al., 2012). However, recurrence or surgical failure is reported in 10% of patients (Duarte et al., 2020). A possible explanation in these patients is that clinical assessment of the NVC responsible for HFS mainly relies on intraoperative findings, judgments, and procedures (Lee et al., 2021; Maroon, 1978; Sekula et al., 2014), and the complex and unusual anatomy of the relationship between the facial nerve and vessels may not have been fully evaluated. The use of magnetic resonance imaging (MRI) allows for the preoperative assessment of NVC (Zhu et al., 2020), enabling the evaluation of its location and severity while simultaneously excluding pathological conditions such as tumors and inflammation. However, NVC is commonly observed on both the symptomatic and asymptomatic sides of patients with HFS, and the exclusive reliance on MRI to pinpoint the causative NVC events for HFS is subject to limitations (El Refaee et al., 2013).

Although NVC in the susceptible portion of the facial nerve was described as extending from the root exit point (RExP) to a few millimeters past the root detachment point (RDP) and was considered the main cause of HFS (Hermier, 2018; Jannetta, 1975; Xue et al., 2021), some studies have revealed that NVC in the distal cisternal portion of the facial nerve also causes HFS (Hatayama et al., 2015; Nomura et al., 2021). Other studies have indicated that factors such as asymmetry of the vertebral arteries (Guan et al., 2011; Park et al., 2015) and symptomatic nerve laterality (Yan et al., 2021) may also play a role in the development of HFS. The etiology of HFSs could be intricate. Furthermore, previous studies often concentrated on patients who underwent microvascular decompression surgery, which lead to the loss of some radiologic characteristics in patients with HFS. A more detailed study of the demographic and radiological characteristics related to HFS is warranted.

This study aimed to identify relevant MRI characteristics associated with HFS occurrence. Additionally, we explored the characteristics of HFS, wherever applicable, to enhance both its understanding and clinical management.

## 2 METHODS

### 2.1 Patients

**FIGURE 1:**
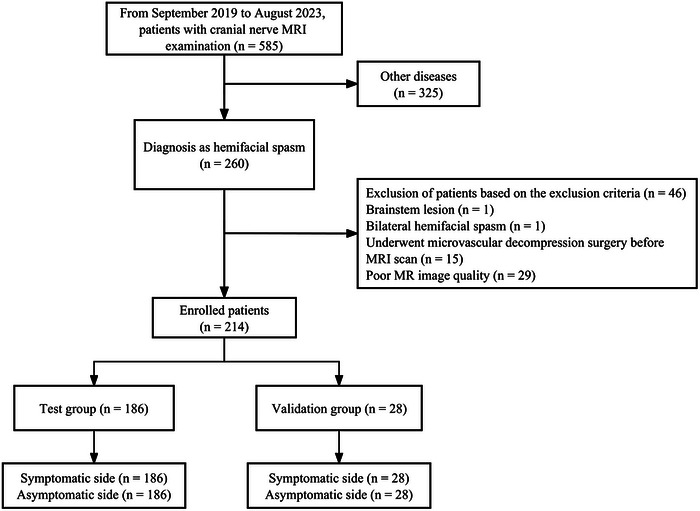
Cohort flowchart for patient selection.

We retrospectively searched our institution's picture achieving and communication system from September 2019 to August 2023 to identify consecutive patients with HFS (Figure 1). The inclusion criteria were (1) patients diagnosed with primary HFS and (2) those who had undergone a dedicated cranial nerve MRI. Patients diagnosed with secondary HFS (e.g., HFS caused by other diseases such as tumors, cysts, or multiple sclerosis), brainstem lesions, bilateral HFS, those who had undergone microvascular decompression surgery before MRI, and those with missing image data or severe motion artifacts influencing the quality of imaging were excluded. The included patients were divided into test and validation groups. This study was approved by the local medical ethics committee, and the need for patient consent was waived because of the retrospective nature of the study.

### 2.2 MRI technique

**FIGURE 2:**
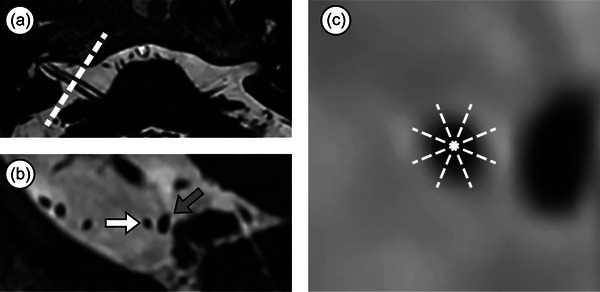
Oblique sagittal reconstructions and orientation schema of the facial nerve. (a) The oblique sagittal plane is orthogonal to the axis of the facial nerve in the cisternal segment in the axial plane. (b) Oblique sagittal plane showing that the rostral is the facial nerve (white arrow) and the caudal is the vestibulocochlear nerve (gray arrow). (c) A localized magnification of the oblique sagittal plane of the facial nerve. The facial nerve is divided into eight equal parts by four dotted lines in a clockwise direction, namely superior, superior–posterior, posterior, inferior–posterior, inferior, inferior–anterior, anterior, and superior–anterior.

The studies were performed using two 3‐T MR scanners (Architect or Discovery; GE Healthcare). MRI sequences included three‐dimensional (3D) fast imaging employing steady‐state acquisition cycled phases (3D‐FIESTA‐C), 3D time‐of‐flight magnetic resonance angiography before and 3 min after the administration of a gadolinium‐chelate contrast agent (Omniscan, GE Healthcare, 0.2 mmol kg^−1^), and 3D gadolinium‐enhanced T1‐weighted sequences. All axial sequences covered the posterior fossa to the basilar artery bifurcation and were acquired parallel to the canthomeatal line. The oblique sagittal plane orthogonal to the axis of the facial nerve in the cisternal segment and oblique coronal plane along the longitudinal axis of the facial nerve were reconstructed using the 3D‐FIESTA‐C sequence (Figure 2).

### 2.3 Imaging analysis

**FIGURE 3:**
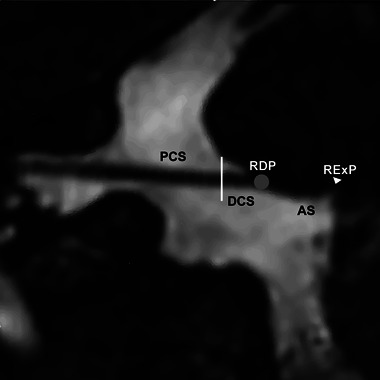
Facial nerve segmentation. Oblique coronal plane along the longitudinal axis of the facial nerve formed by reconstructed of three‐dimensional fast‐imaging employing steady‐state acquisition cycled sequences. The facial nerve stretches from the root exit point (RExP, white arrowhead) and then remains strongly attached to the pontine surface before separating from the brainstem as the attached segment (AS), followed by the root detachment point (RDP), where the nerve detaches from the pons. The proximal cisternal segment (PCS) is the point where the facial nerve extends 3 mm from the RDP. The distal cisternal portion (DCS) is defined as the portion that extends laterally beyond the PCS (white line) up to the porus acusticus.

**FIGURE 4:**
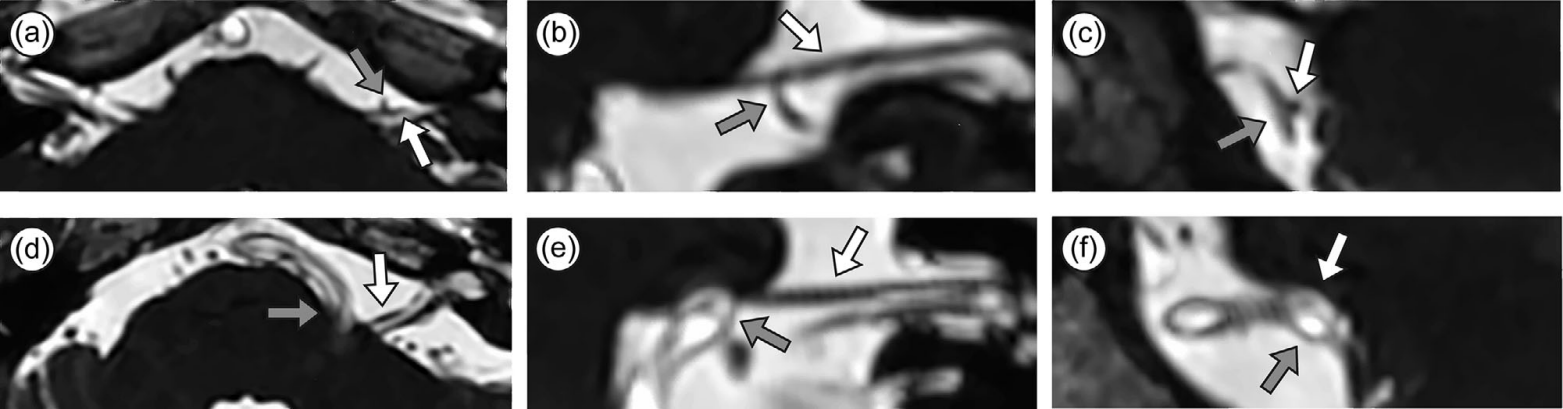
Schema of the degree of neurovascular compression. (a and d) The axial planes of facial nerve; (b and e) oblique coronal planes of facial nerve reconstructed from axial planes; (c and f) oblique sagittal planes of facial nerve reconstructed from axial planes. (a–c) The facial nerve (white arrow) was contacted by the anterior inferior cerebellar artery (gray arrow) contact. Oblique sagittal planes (c) showing the anterior and inferior–anterior of the facial nerve contacted by the offending artery. (d–f) The facial nerve (white arrow) was deformed by the vertebral artery (gray arrow) deform. Oblique sagittal planes (f) showing the inferior portion of the facial nerve deformed by the offending artery.

The MRI examinations were independently evaluated by two radiologists (L.B. and L.C.) with a minimum experience of 5 years, both blinded to the clinical information and symptomatic side. We adopted anatomical terms used by Traylor et al. (2021). The facial nerve was divided into four portions: attached segment (AS), RDP, proximal cisternal segment (PCS), and distal cisternal segment (DCS) (Figure 3). The severity of NVC was categorized as non‐contact (with discernible CSF between the vessel and the facial nerve), contact (the vessel merely touches the facial nerve), and deformity (the vessel displaced the facial nerve) (Figure 4). The facial nerve in both the symptomatic and asymptomatic sides was evaluated for the following variables: presence and severity of NVC in each portion; type of offending vessel, including the anterior–inferior cerebellar artery (AICA), posterior–inferior cerebellar artery (PICA), vertebral artery (VA), superior cerebellar artery, or vein. The orientation of offending vessel on the facial nerve was observed on the oblique coronal plane (Figure 2). If there was more than one NVC in a portion, the highest severity of compression was recorded. Inconsistencies were addressed through a consensus reached after a re‐evaluation of the images.

### 2.4 Diagnostic performance evaluation

The validation group was used to verify whether our findings improved diagnostic performance. One radiologist used the conventional diagnostic method (NVC within the root exit zone of the facial nerve, extending from the RExP to 3 mm lateral to the root entry zone [RDP], has the potential to lead to HFS), and another radiologist used the diagnostic method that was improved basing on our findings to blindly evaluate whether occurrence of HFS in two sides of patients, respectively. The sensitivity, specificity, positive predictive value (PPV), and negative predictive value (NPV) for both the methods, with clinical symptoms as a reference, were calculated.

### 2.5 Statistical analysis

All statistical analyses were performed using SPSS software (version 26.0; IBM Co.) and R software version 2023.03.0. For demographic and clinical characteristics, continuous variables are presented as mean with standard deviation or median with the 25th–75th percentile, as appropriate. Categorical variables are presented as *n* (%). McNemar's test, Chi square test, *t*‐test, or Mann–Whitney test was used appropriately. Cohen's kappa coefficient was used to assess the agreement between two radiologists. The level of agreement was interpreted as follows: slight (0–0.20), fair (0.21–0.40), moderate (0.41–0.60), substantial (0.61–0.80), and almost perfect (0.81–1). The diagnostic performance of two radiologists was assessed at receiver operating characteristic curve analysis, reporting the area under the receiver operating characteristic curve (AUC) using the DeLong method. In the stage of investigating MRI characteristics associated with HFS, we recounted the involvement of each offending vessel as “yes/no” irrespective of the specific segment they affected or the frequency of compression. Treating the DCS, PCS, RDP, and AS with dummy variables, univariate and multivariate conditional logistic regression analyses were performed to identify clinical and radiological characteristics associated with HFS occurrence. All *p*‐values were two‐sided, and all results with *p* ˂ .05 were considered statistically significant.

## 3 RESULTS

### 3.1 Clinical characteristics of the patients

This study included 260 patients with HFS who underwent MRI, of whom 46 were excluded because of the following reasons: brainstem lesions (*n *= 1), bilateral HFSs (*n* = 1), postoperative patients (*n *= 15), missing image data, or severe artifacts (*n *= 29). Ultimately, a total of 214 patients were included, with 186 in the test group and 28 in the validation group. There were no significant differences in demographic and clinical characteristics between the two groups, as indicated in Table 1 (all *p* > .05).

**TABLE 1:** Demographic and clinical characteristics.

Demographic and clinical characteristics	Test group (*n* = 186)	Validation group (*n* = 28)	*p*
Sex			.384^a^
Male	64 (34.4%)	12 (42.9%)	
Female	122 (65.6%)	16 (57.1%)	
Side of HFS			.162^a^
Left	100 (53.8%)	19 (67.9%)	
Right	86 (46.2%)	9 (32.1%)	
Age, years, (mean ± SD)	51.10 ± 11.67	53.21 ± 9.87	.364^b^
Duration, months, (median[IQR])	24[6, 48]	15[6, 36]	.291^c^

Abbreviations: HFS, hemifacial spasm; IQR, interquartile range.

^a^ Chi square test.

^b^ *t*‐test.

^c^ Mann–Whitney test.

### 3.2 Radiological characteristics of the patients

Table 2 compares the MRI characteristics of the symptomatic (*n *= 186) and asymptomatic sides (*n *= 186) in the test group of patients with HFS. Regarding the location of NVC, the PCS, RDP, and AS of the symptomatic nerve were more likely to be in conflict than those on the asymptomatic side (all *p* < .001). In the AS, the contact and deformity ratios on the symptomatic side were 34.4% and 21.0%, whereas on the contralateral side, they were just 16.7% and 1.1%, respectively. In the RDP, the contact and deformity ratios on the symptomatic side were 34.9% and 9.7%, whereas on the contralateral side, they were 10.8% and 0.5%, respectively. In the PCS, the contact and deformity ratios on the symptomatic side were 30.1% and 10.2%, whereas on the contralateral side, they were 23.7% and 0.5%, respectively. In addition, in the DCS, the contact and deformity ratios on the symptomatic side were 46.8% and 5.4%, and on the asymptomatic side, they were 50.0% and 2.7%. Regarding the offending vessel, AICA involvement was present in 145 patients with symptomatic nerves (78.0%) and 108 asymptomatic nerves (58.1%), PICA involvement was observed in 63 symptomatic nerves (33.9%) and 21 asymptomatic nerves (11.3%), and VA involvement was present in 39 symptomatic nerves (21.0%) and 5 asymptomatic nerves (2.7%). Additionally, veins were implicated in 26 symptomatic nerves (14.0%) and in 27 asymptomatic nerves (14.5%). The superior cerebellar artery was not involved in any of the NVC events in our study.

**TABLE 2:** Magnetic resonance imaging (MRI) characteristics of hemifacial spasm patients.

Radiological features	Symptomatic side (*n* = 186)	Asymptomatic side (*n *= 186)	*p*
Location			
DCS			
Contact	87 (46.8%)	93 (50.0%)	.419
Deformity	10 (5.4%)	5 (2.7%)	
PCS			**<.001**
Contact	56 (30.1%)	44 (23.7%)	
Deformity	19 (10.2%)	1 (.5%)	
RDP			**<.001**
Contact	65 (34.9%)	20 (10.8%)	
Deformity	18 (9.7%)	1 (.5%)	
AS			**<.001**
Contact	64 (34.4%)	30 (16.7%)	
Deformity	39 (21.0%)	2 (1.1%)	
Offending vessel			
AICA involvement	145 (78.0%)	108 (58.1%)	**<.001**
PICA involvement	63 (33.9%)	21 (11.3%)	**<.001**
VA involvement	39 (21.0%)	5 (2.7%)	**<.001**
Vein involvement	26 (14.0%)	27 (14.5%)	1.000

*Note*: Statistically significant values are reported in bold. McNemar's test for paired proportions was used to assess differences between the symptomatic and asymptomatic sides.

Abbreviations: AICA, anterior inferior cerebellar artery; AS, attached segment; DCS, distal cisternal segment; PCS, proximal cisternal segment; PICA, posterior inferior cerebellar artery; RDP, root detachment point; VA, vertebral artery.

### 3.3 Clinical and radiologic characteristics associated with the HFS occurrence

Table 3 shows the univariate and multivariate conditional logistic analyses of factors associated with HFS occurrence. Multicollinearity among these components was not a concern in this model, as evidenced by the variance inflation factors for each of them being determined to be less than 1.5. Based on multivariate analysis, deformity in the PCS was significantly correlated with HFS occurrence to non‐contact, with an odds ratio (OR) of 256.58 (95% confidence interval [CI]: 7.34–8972.94, *p* = .002), whereas contact was not (*p* = .233). Compressions in the RDP or AS, whether in the form of contact or deformity, were significantly correlated with HFS occurrence compared to non‐contact. The ORs for contact and deformity in the RDP were 19.98 (95% CI: 5.76–69.36, *p* < .001) and 37.22 (95% CI: 2.15–643.56, *p* = .013), respectively. The ORs for contact and deformity in the AS were 4.99 (95% CI: 1.98–12.55, *p *= .001) and 252.52 (95% CI: 12.41–5138.50, *p *˂ .001), respectively. Furthermore, involvement of the AICA (OR: 5.53, 95% CI: 1.70–18.04, *p* = .005) was significantly correlated with HFS occurrence compared to non‐involvement. However, involvement of the PICA, VA, and laterality did not show a significant correlation with HFS occurrence.

**TABLE 3:** Magnetic resonance imaging (MRI) characteristics associated with hemifacial spasm in univariate and multivariate conditional logistic regression.

Variables	Univariate analysis		Multivariate analysis	
	OR (95% CI)	*p*	OR (95% CI)	*p*
Laterality (“left” as reference)	.86 (.65–1.15)	.305	N/A	N/A
DCS involvement (“non‐contact” as reference)		.383	N/A	N/A
Contact	.94 (.61–1.43)	.754	N/A	N/A
Deformity	2.16 (.65–7.21)	.211	N/A	N/A
PCS involvement (“non‐contact” as reference)		**.004**		**.008**
Contact	1.54 (.95–2.48)	.080	1.78 (.69–4.57)	.233
Deformity	20.50 (2.73–153.69)	**.003**	256.58 (7.34–8972.94)	**.002**
RDP involvement (“non‐contact” as reference)		**.001**		**<.001**
Contact	5.47 (2.84–10.53)	**.001**	19.98 (5.76–69.36)	**<.001**
Deformity	25.35 (3.22–199.41)	**.002**	37.22 (2.15–643.56)	**.013**
AS involvement (“non‐contact” as reference)		**<.001**		**<.001**
Contact	3.77 (2.11–6.75)	**<.001**	4.99 (1.98–12.55)	**.001**
Deformity	28.05 (6.55–120.15)	**<.001**	252.52 (12.41–5138.50)	**<.001**
AICA (“no” as reference)	2.95 (1.75–4.96)	**<.001**	5.53 (1.70–18.04)	**.005**
PICA (“no” as reference)	5.20 (2.64–10.23)	**<.001**	2.72 (.90–8.24)	.077
VA (“no” as reference)	7.80 (3.07–19.79)	**<.001**	2.42 (.45–12.91)	.302
Vein (“no” as reference)	.96 (.54–1.70)	.884	N/A	N/A

*Note*: Statistically significant values are reported in bold.

Abbreviations: AICA, anterior inferior cerebellar artery; AS, attached segment; DCS, distal cisternal segment; OR, odds ratio; PCS, proximal cisternal segment; PICA, posterior inferior cerebellar artery; RDP, root detachment point; VA, vertebral artery.

### 3.4 Findings‐based diagnostic performance

For the assessment of aforementioned radiological characteristics in the validation group, substantial to almost perfect inter‐rater agreement was achieved between the two radiologists. In the evaluation of the occurrence of HFS, both methods achieved a sensitivity of 100%. However, the method improved based on our findings achieved a specificity of 92.9%, surpassing the conventional judgment method's specificity of 64.3%. Additionally, our method achieved a PPV of 93.3%, which exceeded the conventional method's PPV of 73.7% (Table 4). Our method exhibited an AUC value of 0.964, surpassing the conventional method's AUC of 0.821. The difference is statistically significant.

**TABLE 4:** Diagnostic performance of conventional diagnostic methods (radiologist 1) and improved methods based on our findings (radiologist 2) in 28 patients with unilateral hemifacial spasm.

	Sensitivity	Specificity	PPV	NPV	Accuracy	AUC	*p* ^a^
Radiologist 1	28/28	18/28	28/38	18/18	46/56	0.964	.001
	100	64.3	73.7	100	82.1		
Radiologist 2	28/28	26/28	28/30	26/26	54/56	0.821	
	100	92.9	93.3	100	96.4		

Abbreviations: NPV, negative predictive value; PPV, positive predictive value.

^a^ The DeLong method showed that the difference between the two AUCs of radiologists 1 and 2 was statistically significant.

### 3.5 Inclined compression orientation of each offending vessel

**FIGURE 5:**
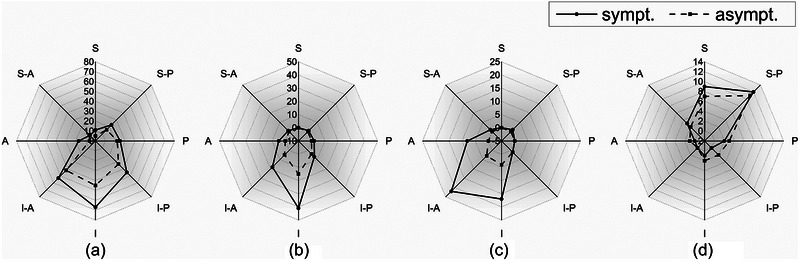
Inclined compression orientation of each offending vessel. The black solid line refers to the offending vessel on the symptomatic side and the black dotted line refers to that in asymptomatic side. The anterior inferior cerebellar artery (AICA) (a) predominantly in the inferior–posterior, inferior, and inferior–anterior positions; the posterior–inferior cerebellar artery (PICA) (b) predominantly in the inferior position; the vertebral artery (VA) (c) predominantly compresses the facial nerve in the inferior–anterior position in both symptomatic and asymptomatic sides; the vein (d) predominantly in the superior and superior–posterior positions.

Figure 5 shows the orientation of the NVC for each offending vessel. The VA predominantly compresses the facial nerve in the inferior–anterior and inferior positions; the AICA predominantly compresses the facial nerve in the inferior–posterior, inferior, and inferior–anterior positions; the PICA predominantly compresses the facial nerve in the inferior position; vein predominantly compress the facial nerve in the superior and posterior–superior positions, respectively. No statistically significant difference was observed between the symptomatic and asymptomatic sides for all vessels (all *p *> .05) (Table S1).

## 4 DISCUSSION

Our blinded study examined the entire facial nerve from the RExP of brainstem to the porous acusticus and vessels, including both arteries and veins, and explored the MRI characteristics associated with HFS occurrence. These findings validated that NVC is more frequently observed on the symptomatic side of patients with HFS and is common on the asymptomatic side as well. Moreover, our study found that deformity in the PCS was significantly independently associated with HFS occurrence, whereas contact was not. Both contact and deformity in the RDP or AS are significantly independently associated with HFS occurrence.

In previous studies, the NVC in susceptible portion is commonly associated with HFS (Hermier, 2018). To our knowledge, our study is the first to divide the susceptible portion of facial nerve into three, including the AS, RDP, and PCS, to analyze the association between these portions and HFS occurrence separately. This study confirmed the previous point and further found that the severity of NVC required for HFS occurrence may vary among different portions. The RDP and AS emerge from the RExP and then firmly attach to the pontine surface before separating from the brainstem. These portions are covered with central oligodendrocyte‐derived myelin without resistance to NVC (Campos‐Benitez & Kaufmann, 2008; Tomii et al., 2003). The PCS, also known as the transition zone, is a transitional portion from central oligodendrocyte‐derived myelin to peripheral Schwann cell‐derived myelin (Alfieri et al., 2012). The peripheral Schwann cell‐derived myelin is considered to be resistant to NVC. We conclude that the partial peripheral Schwann cell‐derived myelin fibers in the PCS may contribute to its mild resistance to NVC, resulting in a high risk of HFS occurrence in cases of high severity of compression. Some anatomical research revealed that the first few millimeters of the AS stretches from the RExP may be covered by the medulla oblongata and also contribute to its mild resistance to NVC (Nomura et al., 2021). Therefore, we treated the RDP, a point rather than a real segment, as a separate portion because the RDP covered by central oligodendrocytes may respond differently to NVC compared to AS and DCS. Although our results indicated that the AS demonstrated a similar response to NVC with the RDP, we propose exercising prudence to consider NVC in the AS as a potential risk factor when it is closely located to RExP. Regarding the DCS, NVC was frequently observed on both the symptomatic (52.2%, 97/186) and asymptomatic sides (52.7%, 98/186). The main portion of facial nerve in the cerebellopontine angle was the DCS. The AICA commonly bifurcates near the facial nerve and gives rise to the internal auditory arteries (Bambakidis et al., 2009; Martin et al., 1980). These characteristics increase the likelihood of NVC in DCS. However, our results were similar to previous findings showing that DCS had no significant role in the development of HFS. It is worth noting that we treated the severity of compression in each portion of the facial nerve as an individual variable. In cases where multiple NVC events occurred in one portion simultaneously, we recorded the most severe degree of NVC. Consequently, a small number of patients may have experienced both contact and deformity within the same portion, potentially resulting in contact being overlooked. This approach was chosen as our primary objective was to investigate the correlation between the severity of compression in each portion and the HFS occurrence, based on radiological findings. In future studies, a more detailed investigation of the relationship between varying severity of NVC in adjacent sites and the development of HFS, utilizing both radiologic and surgical data, can be pursued.

In contrast to the study by Traylor et al. (2021), which reported a deformity of the NVC on the symptomatic side in up to 70.3% of the cases, our findings demonstrated that deformity was only observed in a minority of symptomatic sides, with the highest incidence observed in the AS at 20.9%. We speculate that this could partly be attributed to differences in patient inclusion. Previous studies have primarily focused on patients with HFS who underwent microvascular decompression surgery (El Refaee et al., 2013), whereas our study included both surgically and non‐surgically treated patients. Typically, patients receive medications such as intramuscular botulinum toxin injections before surgery and opt for surgery when nonsurgical treatments are ineffective or when clinical symptoms are severe (Rosenstengel et al., 2012). Excluding those who had only received conservative or drug treatments may introduce potential selection bias and lead to the loss of imaging characteristics in HFS (Zhang et al., 2022). The difference in deformity incidence indicates that the severity of NVC may be related to the seriousness of symptoms. We believe that these findings could improve our understanding of HFS. Further studies are needed to augment the sample size and evaluate this relationship more accurately.

Our study showed that venous compression was detected in 26 patients on the symptomatic side (14.3%) and in 27 patients on the asymptomatic side (19.7%). Previous studies have remained controversial regarding whether NVC by the vein causes clinical symptoms (Naraghi et al., 2007; Rosenstengel et al., 2012; Zhang et al., 2022); our results demonstrated that venous compression was not significantly associated with HFS occurrence. Regarding the offending arteries, although more arterial compression events were observed on the symptomatic side than on the asymptomatic side, multivariate conditional regression analysis revealed that the PICA and VA were not independently associated with HFS occurrence. We contended that their influence on HFS was mediated by NVC severity. These findings suggest that the specific vessel that causes conflict with the facial nerve may not be the determining factor, and clinicians should concentrate on assessing the location and severity of NVC. Based on our findings, a validation study was conducted with another patient group, demonstrating higher specificity and PPV compared to the conventional diagnostic method. Certainly, the role of MRI in the diagnosis of HFS is increasingly prominent. However, it is crucial to note that the diagnosis of HFS still primarily relies on clinical assessment, including the detection of lateral spread of electromyography potentials. Additionally, in clinical practice, NVC events can be observed in the MRI of healthy individuals (Deep et al., 2016). This emphasizes the importance for radiologists, when confronted with NVC events or patients with HFS, to conduct a comprehensive assessment integrating clinical manifestations. It is noteworthy that the objective of this particular validation study is not to promote MRI as the primary diagnostic method for HFS, as clinical symptoms are inherently more intuitive than radiological imaging. Instead, this part of the validation experiment served to confirm that our findings improve the specificity of HFS diagnosis while maintaining diagnostic sensitivity. This suggests that our approach may enhance the accuracy of excluding non‐responsible NVC events and improve the identification of responsible NVC events. We anticipate that these findings will guide radiologists and neurosurgeons toward a more accurate identification of responsible NVC events before surgery. Additional surgical studies are also warranted in the future to further substantiate our findings. However, owing to a thorough evaluation of the severity of patient symptoms and the potential benefits of surgery, some patients with milder symptoms often opt for nonsurgical conservative treatment. As a result, validating the imaging characteristics of this subgroup through surgical findings presents a challenge. Furthermore, our results demonstrated that each offending vessel exhibited preferred compression characteristics. Although these characteristics are mainly associated with the anatomy of the cerebellar regions, to our knowledge, this study presents these findings intuitively for the first time and may have the potential to guide neurosurgeons in performing better surgical procedures, minimizing nerve and vessel injuries.

Although our study revealed that NVC was associated with HFS occurrence under certain conditions, our retrospective cross‐sectional study could not confirm the causality between these factors and the onset of HFS. Some theories point out that focal demyelination of the facial nerve is the main pathogenesis of HFS (Rosenstengel et al., 2012). Luo et al. (2021) used diffusion spectrum imaging (DSI), a high‐resolution fiber tracking technology of MRI, to detect the integrity of the trigeminal white matter in patients with trigeminal neuralgia and demonstrated that the quantitative anisotropy, fractional anisotropy (FA), and general FA of the trigeminal nerve were decreased on the symptomatic side. However, the spatial resolution limits the use of DSI on the facial nerve. The emergence of high‐resolution functional imaging techniques may solve this problem in the future.

This study has several limitations. The inclusion criteria resulted in the absence of surgical verification for MRI characteristics in certain patients. Typically, during microvascular decompression surgery, neurosurgeons can make more intuitive and precise assessments of the location and severity of NVC events in patients with HFS. However, it is worth noting that our study was originally designed to comprehensively assess imaging characteristics in both surgically and nonsurgically treated HFS patients, with the primary goal of advancing our understanding of this disease, and was grounded in the high concordance between MRI and surgical findings and the advantages of unbiased patient selection in minimizing result bias (El Refaee et al., 2013; Jia et al., 2016; Zhang et al., 2022). Nevertheless, this study still faces the challenge of potential inconsistency between imaging findings and the severity observed during surgery (Brinzeu et al., 2018); to obviate this problem, two radiologists were involved in the evaluation. The single‐center design of the study was limited by the sample size, which was based on the number of eligible patients during the study period rather than determined through sample‐based calculations.

## 5 CONCLUSION

This study systematically investigated the relationship between the facial nerve and vessels in patients, including both surgical and nonsurgical, and demonstrated that deformity in the PCS was independently associated with HFS occurrence, whereas contact did not. NVC along the RDP and AS were both independent associated with the occurrence of HFS, and nerve deformity has a higher risk than contact. Our findings validate and further illustrate that different portions of the facial nerve may respond differently to NVC. Radiologists and neurosurgeons should pay close attention to the location and severity of NVC. Moreover, we have demonstrated the specific orientations in which NVC is more likely to occur for each vessel. These findings may significantly enhance our understanding of HFS and provide valuable guidance to neurosurgeons when optimizing surgical procedures, reducing the risk of nerve and vessel injuries.

## AUTHOR CONTRIBUTIONS

**Bo Li**: Conceptualization; methodology; investigation; validation; formal analysis; writing—original draft; writing—review and editing; Visualization; Project administration. **Chun Luo**: Conceptualization; data curation; investigation; writing—review and editing; methodology; visualization; validation. **Yabin Jin**: Software; formal analysis; investigation; writing—review and editing; methodology. **Ying Yi**: Conceptualization; methodology; software; data curation; validation. **Dongliang Cheng**: Methodology; investigation; validation; formal analysis; data curation. **Linwen Huang**: Data curation; formal analysis; software; resources; project administration. **Guofu Wang**: Investigation; visualization; validation; methodology; formal analysis. **Xuguang Zhong**: Investigation; methodology; visualization; validation; formal analysis. **Hai Zhao**: writing—review and editing; investigation; conceptualization; visualization; project administration; supervision. **Mingyong Gao**: Supervision; project administration; conceptualization; methodology; writing—review and editing; visualization; formal analysis; funding acquisition.

### PEER REVIEW

The peer review history for this article is available at https://publons.com/publon/10.1002/brb3.3438.

## Supporting information

Table S1 The orientation of the neurovascular compression for each offending vessel.

## Data Availability

Data supporting the findings of this study are available upon request from the corresponding authors.
