# Supplementary material for: Evaluating magnetic resonance imaging characteristics and risk factors for hemifacial spasm
Source: Brain Behav. 2024 Feb 26;14(2):e3438. doi: 10.1002/brb3.3438 (PMC10897361; doi:10.1002/brb3.3438)
Supplement: Supplementary file 1 — Table S1 The orientation of the neurovascular compression for each offending vessel. [file BRB3-14-e3438-s001.docx]

**Supplementary Table.** **The orientation of the neurovascular compression for each offending vessel.**

|  | AICA | | |  | PICA | | |  | VA | | |  | Vein | | |
| --- | --- | --- | --- | --- | --- | --- | --- | --- | --- | --- | --- | --- | --- | --- | --- |
| Orientation | Sym | Asympt | *p* |  | Sym | Asym | *p* |  | Sym | Asym | *p* |  | Sym | Asym | *p* |
| Superior | 11 | 5 | 0.692 |  | 0 | 0 | 0.785 |  | 0 | 0 | 0.458 |  | 9 | 7 | 0.834 |
| Superior-posterior | 23 | 16 |  |  | 1 | 0 |  |  | 1 | 0 |  |  | 12 | 11 |  |
| posterior | 25 | 22 |  |  | 2 | 0 |  |  | 0 | 0 |  |  | 2 | 3 |  |
| inferior-posterior | 45 | 33 |  |  | 7 | 4 |  |  | 1 | 1 |  |  | 0 | 2 |  |
| inferior | 67 | 45 |  |  | 41 | 15 |  |  | 17 | 4 |  |  | 1 | 2 |  |
| inferior-anterior | 53 | 42 |  |  | 18 | 5 |  |  | 22 | 3 |  |  | 0 | 0 |  |
| anterior | 17 | 6 |  |  | 5 | 0 |  |  | 8 | 0 |  |  | 0 | 1 |  |
| superior-anterior | 8 | 9 |  |  | 1 | 0 |  |  | 1 | 0 |  |  | 3 | 2 |  |

 Statistically significant values are reported in bold. Chi square test was used to assess differences between the symptomatic and asymptomatic side. Abbreviations: AICA, anterior inferior cerebellar artery; PICA, posterior inferior cerebellar artery; VA, vertebral artery; Sym, symptomatic side; Asym, asymptomatic side.
